# Utility of mtDNA-COI Barcode Region for Phylogenetic Relationship and Diagnosis of Five Common Pest Cockroaches

**Published:** 2017-05-27

**Authors:** Saedeh Sadat Hashemi-Aghdam, Golnaz Rafie, Sanaz Akbari, Mohammad Ali Oshaghi

**Affiliations:** 1 Deptartment of Biology, Damghan Branch, Islamic Azad University, Damghan, Iran; 2 Department of Medical Entomology and Vector Control, School of Public Health, Tehran University of Medical Sciences, Tehran, Iran

**Keywords:** Cockroach, mtDNA-COI, PCR-RFLP, Molecular marker, Phylogeny

## Abstract

**Background:**

Cockroaches are of vital importance medically and hygienically as they can disperse human pathogenic agents and are especially responsible for food contamination and spreading of food borne pathogens. In this study, part of mtDNA-COI gene of five common pest cockroaches was tested for diagnostic and phylogenetic purposes.

**Methods:**

We have described barcode region of mtDNA-COI gene of five cockroach species: *Blattella germanica, Blatta orientalis, Periplaneta americana, Shelfordella lateralis*, and *Supella longipalpa,* along with the development of a PCR-RFLP method for rapid detection and differentiation of these health pest species.

**Results:**

The PCR generates a single 710 bp-sized amplicon in all cockroach specimens, followed by direct sequencing. *Alu*I predicted from the sequencing data provided different RFLP profiles among five species. There was a significant intra-species variation within the American cockroach populations, but no genetic variation within other species. Accordingly, phylogenetic analysis demonstrates common monophyly for cockroach families in agreement with conventional taxonomy. However *S. longipalpa* (Ectobiidae) diverged as an early ancestor of other cockroaches and was not associated with other Ectobiidae.

**Conclusion:**

The PCR-RFLP protocol might be useful when the conventional taxonomic methods are not able to identify specimens, particularly when only small body parts of specimens are available or they are in a decaying condition. mtDNA-COI gene shows potentially useful for studying phylogenetic relationships of Blattodea order.

## Introduction

Cockroaches are considered one of the most successful groups of animals. Because cockroaches are so adaptable, they are almost found around the world with most species living in tropical and equatorial regions (Schal and Hamilton 1990). Cockroaches are synanthropic and endophilic, exhibit communicative behavior, attracted to dirt and filth that could contain human pathogens and human food products, and harbors these pathogens. Cockroaches are present in homes, groceries, food warehouses, restaurants, hotels, hospitals as well as in sewer systems and rubbish bins. These biological characters and their ecological association with humans positioned them in the list of dirty species by FDA as the greatest health hazard risk for transmitting human diseases through food products (Olsen et al. 2001, Sulaiman et al. 2011). Cockroaches may transmit four strains of poliomyelitis virus, viruses of encephalitis, yellow fever, coxsakie, and the eggs of seven species of pathogenic ringed worms. Moreover, twelve species of adult cockroaches are known as intermediate host for invertebrates, some species of bacteria including: *Salmonella*, *Staphylococcus*, *Streptococcu*s, *Escherichia coli*, *Proteus*, *Klebsiella*, *Serratia* and some protozoa such as: *Giardia*, *Balantidium*, *Entamoba histolytica*, *Trichomonas* and some of the pathogenic fungi such as *Aspergillus*.

Cockroaches coexist with approximately 150 species of bacteria, 60 species of fungi, six species of yeast, 90 species of protozoa, 45 species of pathogenic ringed worms and some of the other worms (Pai et al. 2003, Mlso et al. 2005, Salehzadeh et al. 2007, Sookrung et al. 2008, Karimizarchi and Vatani 2009, Fakoorziba et al. 2010, Akbari et al 2014). They also are a principal contributor to allergies (Arruda et al. 2001, Arlian 2002). In most regions of the world, residual insecticide sprays are applied into cracks and crevices of filthy places to control cockroaches (Limoee et al. 2006).

Based on the current classification, cockroaches belong to the order Blattodea that comprises three superfamilies of Corydioidea, Blaberoidea, and Blattoidea (Beccaloni and Eggleton 2013). Overall, 7570 living species of Blattodea are currently recognized, of which 4641 are cockroaches (Beccaloni 2007) and 2929 are termites (Krishna et al. 2013). About 30 out of 4641 cockroach species are more associated with human habitations where some species such as *Periplaneta americana* (American), *B. germanica* (German), *B. orientalis* (Oriental) are well known as pests (Valles et al. 1999, Schal and Hamilton 1990). In addition, *Shelfordella lateralis* (Turkestan) and *Supella longipalpa* (Brown-banded) are among the rapidly replacing known pest species in some places of the world. *Shelfordella lateralis* is well distributed in central Asia, the Caucasus Mountains, and northeastern Africa. Recently it has been rapidly replacing the common oriental cockroach in urban areas of the southwestern USA (Kim and Rust 2013) and north of Iran (Oshaghi, personnel observation). *Supella longipalpa* (Brown-banded) is a worldwide pest (Charlton et al. 1993) and one of the most recent cockroaches has been gradually replacing the common German cockroach and to form breeding colonies in many parts of the world including Iran (Laddoni, personnel observation).

Cockroach species identification is traditionally based on morphological characteristics, however, morphology is not always a suitable method for these types of studies and sometimes it suffers from deficiencies particularly when only small body parts of the specimen are available or they are in a decaying condition. Consequently, today, in addition to morphological, molecular markers, specifically DNA-based molecular markers are being widely used in animals systematic. Molecular data contributes to resolve the systematic issues in different organisms including cockroaches (Lazebnaya et al. 2005, Mukha et al. 2007, Pechal et al. 2008, Hashemi-Aghdam and Oshaghi 2014). However, the relationships within Blattodea order are still uncertain in the literature.

Studying of the barcode region of cytochrome oxidase subunit I gene of mitochondrial DNA (mtDNA-COI) has been proposed as a modern systematic tool applied in evolutionary and population study on species delimitation and taxonomy. This gene is one of the largest genes in the metazoan mitochondrial genome and its adaptability is more than the other mitochondria genes. It is also one of the most important molecular markers used for molecular taxonomy and systematic of living things and microorganisms (Hebert et al. 2003, Karimian et al. 2014). Of different segments of the COI gene, DNA barcode is selected and used as a standardized region in molecular systematic in literature. In fact, barcoding region provides a common source of DNA sequence for identification and taxonomy of organisms (Hollingsworth et al. 2011), whereby scientists can compare living organisms. As of February 2013, the Barcode of Life Data systems database (http://www.boldsystems.org) included almost 2,000,000 barcode sequences from over 160,000 species of animals, plants and fungi.

In this study, we designed a PCR-RFLP assay from the barcode region of mtDNA COI sequences generated from five cockroach species, and attempted to infer the phylogenetic position of those species in the Blattodea more reliably. This diagnostic and inferring phylogeny might be a valuable tool for cockroach species identification for entomological studies and control measures strategies.

## Materials and Methods

### Sample collection and DNA extraction

*Periplaneta americana*, *B. orientalis*, *B. germanica*, *Sh.lateralis* and *Su. longipalpa* were trapped between March and August 2012–2013 from different locations such as dwelling, hospital, confectionary, park and insectarium in towns located in center, north and northwest of Iran using live-catch trap, box matches and hand catch. The specimens were anesthetized in cold and then preserved in 70% alcohol. Details of the specimens used in this study are described in Table 1. The specimens were morphologically identified according to the available identification keys (Pratt and Stojanovich 1966, Cochran 1999). Fifty-seven different specimens belong to the five species (comprising 14 American cockroaches, 17 German cockroaches, ten Brown-banded cockroaches, eight Oriental cockroaches, and eight Turkistan cockroaches) were used. Genomic DNA was extracted from the hind leg (femur) for larger cockroaches whereas for small ones such German and Brown-banded cockroaches the entire body was used. For DNA extraction, the cockroach femur or entire body was isolated and dried at 37 °C in an incubator. Samples were subsequently placed in liquid nitrogen for 2–5 min and then homogenized with autoclaved glass pestle. DNA was extracted from the resultant homogenate using the method described by Collins et al. (1987) and stored at −20 °C until used.

**Table 1.** Details of sequence data used for cockroach phylogenetic analysis in this study. NS: Not stated

**Species**	**Common name**	**Family**	**Accession** number	**Origin**	**Reference**
***Cryptocercus relictus***	Relict	Cryptocercidae	JX144941	NS	Direct Submission
***Periplaneta japonica***	Japanese	Blattidae	JQ350708	NS	Direct Submission
***Periplaneta fuliginosa***	Smoky brown	Blattidae	AB126004	NS	Direct Submission
***Periplaneta americana***	American	Blattidae	JQ267476	Iran: Kashan	This study
***Periplaneta americana***	American	Blattidae	JQ267480	Iran: Tabriz	This study
***Periplaneta americana***	American	Blattidae	JQ350707	NS	Direct Submission
***Pelmatosilpha guyanae***	N.S.	Blattidae	EU253833	NS	Direct Submission
***Blatta orientalis***	Oriental	Blattidae	JQ267490-JQ267492	Iran: Tabriz	This study
***Blatta orientalis***	Oriental	Blattidae	EU253827	NS	Direct Submission
***Shelfordella lateralis***	Turkestan	Blattidae	JQ267493-JQ267494	Iran: Assalem	This study
***Shelfordella lateralis***	Turkestan	Blattidae	JQ267495	Iran: Tehran	This study
***Phyllodromica iberica***	N.S.	Ectobiidae	AM600690	Spain: Zaragoza	Direct Submission
***Phyllodromica subaptera***	N.S.	Ectobiidae	AM600683	Spain: Granada	Direct Submission
***Parcoblatta pensylvanica***	Pennsylvania wood	Ectobiidae	GU013646	Canada:Ontario	Direct Submission
***Blattella bisignata***	Double-striped	Ectobiidae	JX233805	NS	Direct Submission
***Blattella germanica***	German	Ectobiidae	JQ267496-JQ267498	Iran: Tehran	This study
***Blattella germanica***	German	Ectobiidae	EU854321	China	Direct Submission
***Supella longipalpa***	Brown-banded	Ectobiidae	KJ787108	Iran: Tehran	This study
***Supella longipalpa***	Brown-banded	Ectobiidae	EU253834	NS	Direct Submission
***Eupolyphaga sinensis***	N.S.	Corydiidae	JF700164	NS	Direct Submission
***Polyphaga sp***	N.S.	Corydiidae	JQ267475	Iran: Tehran	Direct Submission
***Therea petiveriana***	Desert	Corydiidae	EU253835	NS	Direct Submission
***Myzus persicae***	Peach-potato aphid	Aphididae	JX844381	China	Direct Submission

### PCR-direct sequencing

A fragment of 710bp of the COI mitochondrial region designated as the barcoding region was amplified by PCR using primers 5′-TTAAACTTCAGGGTGACCAAAAAATCA-3′ (HCO2198) and 5′-G GTCAACAAATCATAAAGATATTGG-3′ (LCO1490) (Folmer et al. 1994). PCRs were carried out in 20μL reaction using 2μL genomic DNA. The PCR reaction consisted of 2μL PreMix (iNtRON®, South Korea), and 18μL of a solution containing 200 nM each primer, and 2μL (50 ng) genomic DNA. The PreMix is a premixed solution containing HotStarTaq DNA polymerase, PCR buffer, and deoxynucleoside triphosphates (dNTPs), with a final concentration of 1.5mM MgCl2 and 200 mM each dNTP. The reactions were run with an initial denaturation of 2 min at 94 °C and then followed first by 5 cycles at 94 °C for 40 sec, 45 °C for 40 sec, 72 °C for 1 min and then 35 cycles at 94 °C for 40 sec, 51 °C for 40 sec, 72 °C for 1 min, with a final extension 72 °C for 5 min in a Peqlab thermocycler machine. The PCR products were electrophoresed on 1% agarose gel with TAE buffer (40mM Tris-acetate and 1mM EDTA, pH 8.0), and stained with Ethidium bromide (EtBr) at a concentration of 0.5μg/ml, along with a 100-bp ladder (SinaClon, Iran).

### Nucleotide sequencing and phylogenetic analysing

The amplified products of mtDNA COI gene for individuals of the five cockroach species were sequenced bi-directionally and consensus nucleotide sequences were obtained. Multiple alignments of the nucleotide sequences were performed using ClustalW2 program (http://www.ebi.ac.uk/Tools/msa/clustalw2). BLAST (http://blast.ncbi.nlm.nih.gov/Blast.cgi) software was used in order to confirm the accuracy of sequences, discovering their identity and rate of similarity with available data in GenBank. The obtained sequences in this study plus a subset of mtDNA-COI sequences of other cockroach species available in GenBank were used for phylogenetic analysis. The sequence data contained 16 species belonged to four families of Cryptocercidae, Blattidae, Ectobiidae (Blattellidae) and Corydiidae (Polyphagidae) (Table 1). The sequence of *Myzus persicae* (Accession No. JX844381) was used as an out-group. Phylogenetic tree inferred using the neighbour-joining method embedded in ClustalW2 program and plotted using Software program Tree view (http://taxonomy.zoology.gla.acuk/rod/rod.html).

### PCR-RFLP

**Fig. 1.**
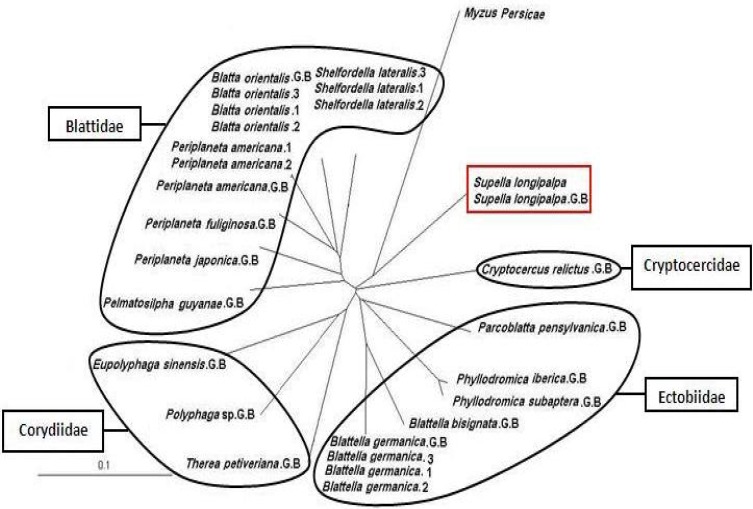
Phylogenetic tree obtained by comparing 630 bp sequences of mtDNA-COI gene of cockroaches. G.B: GenBank. *Myzus persicae* with Accession No. JX844381 is used as outgroup

Based on sequence variation among the five species, AluI restriction enzyme was selected by manufacturer’s NEBcutter V2.0 program (http://tools.neb.com/nebcutter) and a physical map provided (Fig. 1B). For restriction fragment assays, 15μL of the PCR products was digested in a 25μL reaction mixture containing 10 U of AluI (Vivantis) and 2.5μL of the appropriate restriction buffer at 37 °C overnight, following the instructions of manufacturer. The digested products were fractionated on a 2.5% agarose gel and visualized by ethidium bromide staining under ultraviolet light.

## Results

### mtDNA-COI gene and phylogenetic analysis

Result of PCR amplification revealed that the primers could amplify a unique 710bp fragment of the COI gene for all of the five cockroach species. Of the 57 PCR amplified products of the specimens, the mtDNA-COI nucleotide sequences were generated for 26 specimens. Of these, 14 sequences were generated for *P. americana*, three sequences each for *B. germanica*, *S. lateralis*, *S. longipalpa*, and *B. orientalis* specimens. The nucleotide sequences have been deposited in the GenBank database under accession nos. JQ267476 to JQ267498 and KJ787108. On average, a high A-T content (65.7%) was evident in the amplified mtDNA-COI gene fragment of the species. However, the A-T contents did not show significant variations at the intra- or inter-species levels.

A significant inter-species genetic diversity was observed among the five cockroach species characterized at the mtDNA-COI gene. The most divergence rate (22.26%) was observed between *S. longipalpa* and *P. americana* and the least rate (11.57%) was seen between American and Oriental cockroaches. However, the most genetic difference among the species was lowered to 0.0–9.1% at amino acid level indicating that most of the substitutions were silent or synonymous (Table 2). Generally, the brown-banded cockroaches with the lowest similarity rate at DNA (80%) and amino acid (92.86%) levels were the most diverged species among the species (Table 2). Additionally, based on mtDNA molecular clock (2% per MY), *S. longipalpa* is suggested to have diverged from other species about 10 MY. At the intra-species level, a considerable genetic polymorphism (2.62%) was detected among the American cockroach populations whereas no genetic variation has been noticed within other species.

**Table 2.** Genetic similarity rates of mtDNA-COI gene among five cockroach species of *P. americana* (American), *B. orientalis* (Oriental), *S. lateralis* (Turkestan), *B. germanica* (German), and *S. longipalpa* (Brown-banded) at DNA (648 bp, left and below asterisks) and amino acid (210 bases, right and above asterisks) levels

	**American**	**Oriental**	**Turkestan**	**German**	**Brown-banded**
**American**	***	98.57	100	96.19	92.38
**Oriental**	88.43	***	98.57	95.71	91.90
**Turkestan**	87.65	88.27	***	96.19	92.38
**German**	82.10	83.49	82.56	***	92.86
**Brown-banded**	77.74	80.53	80.53	79.44	***

Phylogenetic relationships among 16 species belonged to four cockroach families Cryptocercidae, Blattidae, Ectobiidae (Blattellidae) and Corydiidae (Polyphagidae) were estimated based on the 648bp DNA sequences of the mitochondrial cytochrome oxidase subunit I (COI) gene. A cladogram inferred using the neighbor-joining method indicated monophyly of each of the four families (Fig. 1).

However, *S. longipalpa* of Ectobiidae (Blattellidae) was diverged early and formed a separate branch distinct from other members of Ectobiidae. The phylogenetic tree showed that all species of that Blattidae grouped together and formed a main clad. In addition, they were sister group of Ectobiidae (Blattellidae). The cladogram suggested that *S.longipalpa* was the basal species and the early ancestor of other cockroaches. Following *S. longipalpa*, Cryptocercidae was the basal families of other cockroaches.

### PCR-RFLP

**Fig. 2.**
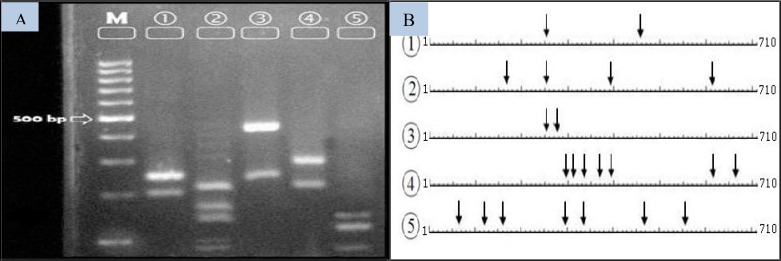
Profile of *Alu*I PCR-RFLP (A) and physical map (B) of mtDNA-COI (barcode region) for five cockroach specis.1: *Shelfordella lateralis*, 2: *Periplaneta americana*, 3: *Blatta orientalis*, 4:*Blattella germanica*, 5: *Supella longipalpa*

Based on nucleotide sequence analysis of the mtDNA-COI gene, a PCR-RFLP assay was developed for the diagnosis and differentiation of the five cockroach species. It was accomplished by digesting the PCR product (710bp) with *Alu*I restriction enzymes. *Alu*I cuts the PCR–amplified mtDNA-COI fragment of the five species at unique positions: at 251 and 455 to produce three bands of 251, 204 and 255bp for *S. lateralis*, at 167, 251, 392, and 612 to produce five bands of 167, 84, 141, 220 and 98bp for *P. americana*, at 251 and 275 to produce three bands of 251, 24, and 435bp for *B. orientalis*, at seven restriction sites to produce eight bands of 296, 15, 24, 33, 24, 220, 50 and 48bp for *B. germanica*, at seven cut sites and therefore produces eight bands of 65, 54, 39, 138, 39, 132, 87 and 156 bp for *S. longipalpa* (Fig. 2). Although some bands had approximate molecular weights (such as 255 and 251bp for *S. lateralis* and 132 and 138bp for *S. longipalpa*) or the bands with less than 100bp length were not easily visible in agarose gel, still a distinctive and visible RFLP profile with *Alu*I restriction enzyme was obvious after restriction digestion for each of these five species (Fig. 2A).

## Discussion

In this study, we developed a PCR-RFLP method and defined a unique restriction enzyme (*Alu*I) for the first time that can rapidly detect and differentiate the five pest species of cockroaches: *P. americana*, *B. orientalis*, *B. germanica*, *Sh. lateralis*, and *S. longipalpa*. Using a simple conventional PCR and just one single restriction enzyme (*Alu*I) for differentiating of five cockroach species is the advantages of this method in comparison with the only previous study that used two restriction enzymes and a more complicated nested-PCR for discrimination of four cockroach species (Sulaiman et al. 2011). PCR-RFLP technique is a very cheap and rapid method for species identification of many organisms. This molecular assay was used to identify many species of organisms such as rodents (Oshaghi et al. 2011a), parasites (Oshaghi et al. 2009, 2010, 2011b), *Anopheles* (Oshaghi et al. 2008, Mehravaran et al. 2011) and to determine blood type within insect guts (Chavshin et al. 2006, Maleki-Ravasan et al. 2009). This will provide a cost-effective solution that so many specimens can be studied with no need to provide DNA sequencing. In this method, using a set of conserved primers, a tiny amount of DNA can be amplified and then be identified by one restriction enzyme that overcomes lack of ample amount of cockroach body parts. In addition, this method can be used to determine identity of museum specimens, unknown samples and malformed specimens or residues of cockroach body parts in food samples.

Analysis of the data revealed a high inter-species variation (11.56–22.26%) in the barcoding region of mtDNA COI gene across these five cockroach species, suggesting the gene fragment is a good molecular marker for the development of a diagnostic tool for the detection of food pests in food samples. DNA barcoding has been used as a shared resource of DNA sequences for identification and taxonomic clarification of many organisms, including birds (Hebert et al. 2004), sea turtles (Vargas et al. 2009), eutherian mammals (Luo et al. 2011), fishes (Mabragana et al. 2011) and insects (Barrett and Hebert 2005, Smith et al. 2006, Wilson 2010). In the light of DNA barcoding, researchers can compare different kinds of organisms and have access to their genetic information providing evidence of interpreting systematic situation. Hebert et al. (2003) proposed the use of COI gene as an original source for species identification in the animal kingdom. Additionally, they demonstrated that species-level assignments could be obtained by creating a comprehensive COI. In addition to COI, many researchers used various genes including rRNA (Kambhampati et al. 1995, Mukha et al. 2000, Lazebnaya et al. 2005), COII (Maekawa and Matsumoto 2000), ETS (Mukha et al. 2002, Mukha et al. 2005), NTS (Mukha et al. 2007), ITS1 (Pechal et al. 2008) and complete mitochondrial genome (Yamauchi et al. 2004, Zhang et al. 2010, Cameron et al. 2012, Xiao et al. 2012, Chen 2013) for systematic classification of different species of cockroaches.

The inferred relationships among cockroach families (*S. longipalpa* + (*S. longipalpa* + (Blattidae + (Cryptocercidae + (Corydiidae + Ectobiidae)))) is partly in agreement with some previously published analyses (Kambhampati 1995, Maekawa and Matsumoto 2000, Lo et al. 2007). Furthermore, *S. longipalpa* has been introduced as the early ancestor of other cockroaches by the phylogenetic trees inferred from both amino acid and DNA sequence data where it did not group with Ectobiidae or indeed nest within cockroaches. It is in controversy with earlier morphology-based phylogenetic hypotheses as well as molecular study using a combination of four mitochondrial (16S and COI+COII) and nuclear (18S and 28S ribosomal subunits) loci indicating monophyletic topology of Blaberoidea superfamily (Djernæ et al. 2002). A wider sampling of Blaberoidea including Blaberidae is highly required in order to test the topological situation of *S. longipalpa*.

## Conclusion

The results of this study demonstrate the utility of the mtDNA-COI gene as a valuable and powerful molecular marker in unraveling medically important cockroach species, particularly when morphological characters are subtle. Moreover, the mtDNA-COI gene shows potentially advantageous for understanding phylogenetic relationships in this order.
